# Inhibition of Stearoyl-CoA desaturase 1 reverts BRAF and MEK inhibition-induced selection of cancer stem cells in BRAF-mutated melanoma

**DOI:** 10.1186/s13046-018-0989-7

**Published:** 2018-12-17

**Authors:** Maria Elena Pisanu, Marcello Maugeri-Saccà, Luigi Fattore, Sara Bruschini, Claudia De Vitis, Eugenio Tabbì, Barbara Bellei, Emilia Migliano, Daniela Kovacs, Emanuela Camera, Mauro Picardo, Ziga Jakopin, Claudia Cippitelli, Armando Bartolazzi, Salvatore Raffa, Maria Rosaria Torrisi, Franco Fulciniti, Paolo A. Ascierto, Gennaro Ciliberto, Rita Mancini

**Affiliations:** 1 Department of Clinical and Molecular Medicine, Sapienza University of Rome, 00161 Rome, Italy; 2 Division of Medical Oncology 2, IRCSS Regina Elena National Cancer Institute, 00144 Rome, Italy; 3 Preclinical Models and New Therapeutics Agents Unit, IRCSS Regina Elena National Cancer Institute, 00144 Rome, Italy; 4 Department of Experimental and Clinical Medicine, Magna Graecia University of Catanzaro, 88100 Catanzaro, Italy; 5 Laboratory of Cutaneous Physiopathology and Integrated Center of Metabolomics research, San Gallicano Dermatologic Institute, IRCSS, 00144 Rome, Italy; 6 Department of Plastic and Reconstructive Surgery, San Gallicano Dermatologic Institute, IRCSS, 00144 Rome, Italy; 7 Faculty of Pharmacy, University of Ljubljana, Ljubljana, Slovenia; 8 Pathology Research laboratory, Sapienza University, Sant’Andrea Hospital, 00189 Rome, Italy; 9 Cellular Diagnostics Unit, Sapienza University, Sant’Andrea Hospital, 00189 Rome, Italy; 10 Istituto Cantonale di Patologia, Servizio di Citologia Clinica, 6600 Locarno, Switzerland; 11 Melanoma, Cancer Immunotherapy and Development Therapeutics Unit, Istituto Nazionale Tumori IRCCS Fondazione “G. Pascale”, 80131 Naples, Italy; 12 Scientific Directorate, Istituto Nazionale Tumori IRCSS Regina Elena, 00128 Rome, Italy; 13 Present Address: High Resolution NMR Unit, Core Facilities, Istituto Superiore di Sanità, 00161 Rome, Italy

**Keywords:** Lipid metabolism, Melanoma cancer stem cells, BRAF, MEK inhibitors

## Abstract

**Background:**

Combination therapy with BRAF and MEK inhibitors significantly improves survival in BRAF mutated melanoma patients but is unable to prevent disease recurrence due to the emergence of drug resistance. Cancer stem cells (CSCs) have been involved in these long-term treatment failures. We previously reported in lung cancer that CSCs maintenance is due to altered lipid metabolism and dependent upon Stearoyl-CoA-desaturase (SCD1)-mediated upregulation of YAP and TAZ. On this ground, we investigated the role of SCD1 in melanoma CSCs.

**Methods:**

SCD1 gene expression data of melanoma patients were downloaded from TCGA and correlated with disease progression by bioinformatics analysis and confirmed on patient’s tissues by qRT-PCR and IHC analyses. The effects of combination of BRAF/MEKi and the SCD1 inhibitor MF-438 were monitored by spheroid-forming and proliferation assays on a panel of BRAF-mutated melanoma cell lines grown in 3D and 2D conditions, respectively. SCD1, YAP/TAZ and stemness markers were evaluated in melanoma cells and tissues by qRT-PCR, WB and Immunofluorescence.

**Results:**

We first observed that SCD1 expression increases during melanoma progression. BRAF-mutated melanoma 3D cultures enriched for CSCs overexpressed SCD1 and were more resistant than 2D differentiated cultures to BRAF and MEK inhibitors. We next showed that exposure of BRAF-mutated melanoma cells to MAPK pathway inhibitors enhanced stemness features by upregulating the expression of YAP/TAZ and downstream genes but surprisingly not SCD1. However, SCD1 pharmacological inhibition was able to downregulate YAP/TAZ and to revert at the same time CSC enrichment and resistance to MAPK inhibitors.

**Conclusions:**

Our data underscore the role of SCD1 as prognostic marker in melanoma and promote the use of SCD1 inhibitors in combination with MAPK inhibitors for the control of drug resistance.

**Electronic supplementary material:**

The online version of this article (10.1186/s13046-018-0989-7) contains supplementary material, which is available to authorized users.

## Background

Over the past years, major breakthroughs have been made in the medical treatment of melanoma. The advent of molecular targeted agents and immune checkpoint inhibitors consistently changed the treatment of patients with advanced melanoma, leading to an unprecedented improvement of survival outcomes [1, 2]. Randomized phase III trials demonstrated that the BRAF inhibitors namely vemurafenib and dabrafenib achieve an efficient targeting of BRAF-mutated melanoma [3]. Given that functional evidence pointed to continued activation of the MAPK pathway as a way cancer cells exploit to overcome the roadblock imposed by BRAF inhibitors, combining BRAF and MEK inhibitors resulted in improved clinical outcomes compared to single agent BRAF-directed therapy [4–6]. Despite these therapeutic successes, melanoma cells acquire the ability to withstand combined BRAF and MEK inhibition. A number of molecular mechanisms, eventually co-existing, have been proposed in the attempt of explaining how melanoma cells adapt to prolonged inhibition of BRAF and MEK. Currently, evidence converge on the functional or mutational reactivation of the MAPK pathway or, alternatively, activation of parallel signalling avenues [7–11]. Adding further complexity to this picture is the existence, within the same tumour, of distinct cell clones relying on different survival pathways.

A concept that acquired increased attention over the past two decades is the existence of an uncommon subpopulation of tumour cells with distinctive features, defined as cancer stem cells (CSCs). This definition is rooted into their ability to self-renew and capability to propagate the tumour upon xenotransplantation in immunocompromised mice [12–14].

The development of dedicated assays for functional characterization of CSCs allowed to clarify two central tenets of CSCs biology. First, the CSC state is not a fixed condition, but rather a condition that can be acquired when cells are exposed to specific stimuli deriving from the microenvironment (e.g. hypoxia, low pH, epithelial-mesenchymal transition, cytokines) [15–19].

Second, CSCs are intrinsically resistant to cytotoxic therapies, such as chemotherapy and radiotherapy, plausibly as result of their slow replication kinetics and an extreme efficiency of the molecular machinery deputed to repair genetic lesions [20, 21]. Indeed, while cytotoxic agents eliminate more differentiated, quickly dividing cells, the CSC pool expands in the attempt of replacing dying tumour cells. On this ground, an efficient targeting of CSCs has been advocated as essential for obtaining long-lasting tumour remission. When considering targeted agents, CSCs seem to be only marginally sensitive to some therapeutics, while being vulnerable to the abrogation of other signalling avenues that are instrumental for their survival [22–24]. Recently, we and others have reported that CSCs maintenance is due to an altered metabolic status, characterized by a larger pool of monounsaturated fatty acids (MUFAs), generated by the activity of the Stearoyl-CoA desaturase 1 (SCD1) [25–32]. In previous studies, we demonstrated that SCD1 is a key factor for lung CSCs, and that its inhibition selectively kills CSCs acting synergistically with chemotherapy [25–28, 32]. Further corroborating this notion, a wave of studies demonstrated that SCD1 confers malignant traits in arrays of experimental models, spanning from ovarian and thyroid cancers to endometrial and lung carcinomas [30, 32–35]. Remarkably, SCD1 is molecularly intertwined with canonical pathways regulating the CSC pool, such as the Hippo transducers YAP/TAZ, Wnt and Nf-Kb [30, 32–35]. Even though SCD1 and, to a broader extent, lipid metabolism is emerging as a central enzymatic node in controlling CSC fate, evidence is still lacking on the connection between SCD1 and resistance to pharmacological inhibition of the MAPK pathway in melanoma.

Herein we analysed the correlation between SCD1 expression and melanoma progression, documenting the nexus between SCD1 and tumour aggressiveness. This prompted us to hypothesize that SCD1 may also be involved in drug resistance. On this ground, we investigated the consequences of targeting BRAF and MEK, alone or in combination, in 3D and 2D in vitro models of melanoma. With this approach, we observed that melanoma CSCs are able to endure targeted agents, and that this process is tied to an increased activity of SCD1. Consistently, pharmacological inhibition of SCD1 efficiently targeted melanoma CSCs and attenuated YAP/TAZ activity, partly reverting their resistance to BRAF and MEK inhibitors.

## Methods

### Reagents and plasmids

2-methyl-5-(6-(4-(2-(trifluoromethyl)phenoxy)piperidin-1-yl)pyridazin-3-yl)-1,3,4-thiadiazole (MF-438) was kindly provided by Ziga Jakopin. Vemurafenib (BRAF inhibitor) and Binimetinib (alias MEK162, MEK inhibitor) were obtained from Selleck Chemicals. Chloroform (CHCl3), methanol (CH3OH), hexane, isopropanol, and bis(trimethylsilyl)-trifluoacetamide (TMS) were of MS grade and were purchased from Merck (Darmstadt, Germany). Butylated hydroxytoluene (BHT), K2SO4, and HCl were purchased from Sigma–Aldrich, (Buchs, Switzerland). Complementary DNA for human SCD1 was cloned into pCDNA3 as described in Noto et al. [26].

### Cell cultures

Established human melanoma cancer cells A375, M14, WM115, LOXIMVI, WM266 and M14-R were obtained as previously described [36]. Cells in adherent condition were cultured in RPMI-1640 (Sigma, St. Louis, MO, USA) supplemented with 10% FBS (Sigma, St. Louis, MO, USA) at 37 °C in an atmosphere of humidified air with 5% CO2.

Primary cancer cell lines Mel 26, Mel 29, Mel 35, Mel 66, Mel 67 and normal melanocytes #1, #2, #3, #4 were obtained from patients as described in Kovacs et al. [37]. Briefly melanoma cells were isolated from specimens obtained from patients enrolled by the Melanoma Unit of the San Gallicano Dermatologic Institute, Istituti Fisioterapici Ospitalieri (IFO) after patients gave written informed consent. Institutional Research Ethics Committee (IFO), approval was obtained to collect samples of human material for research. The tissue was manually crumbled in small pieces and then incubated with collagenase 0.35% for 45 min at 37 °C, centrifuged, resuspended and grown in OptiMEM (Life Technologies, Invitrogen, Milan, Italy) medium containing 10% fetal bovine serum and antibiotics. To maintain the integrity of collections, all the primary cell lines were maintained in culture no more than passages 2-12th. All cells were routinely checked for mycoplasma contamination and analysed for morphology.

### Sphere formation

Sphere propagation assays were performed as previously described [27, 38]. Briefly, single-cell preparation (1000 cells/well) of stable and primary cell lines were suspended in an appropriate amount of sphere-forming medium (serum-free DMEM/F12 supplemented with bEGF, EGF, insulin, glucose, heparin, (Sigma, St. Louis, MO, USA), B27 (Gibco, Invitrogen, Carlsbad, CA, USA)), and plated into a 96-well ultra-low adherent plate (Costar, USA) to form spheres. The documentation of images and evaluation of sphere-forming efficiency were performed on 4 days, or 7 days, as specified.

An average of 8–10 fields were used for these measurements. Sphere-forming efficiency (%) was determined by dividing the number of spheres formed by the original number of seeded cells. The quotient was then multiplied by 100.

### Drug treatments

The determination of IC50 was performed as previously described [28]. Briefly, a dilution series of 3-fold increments of BRAFi/MEKi (0.007–20 μM) or MF-438 (0.007–50 μM), were prepared in sphere medium (or in RPMI-1640 for clonogenic and for 3-(4,5-dimethylthiazol-2-yl)-2,5-diphenyltetrazolium bromide (MTT) assay). Melanoma cells at a density of 1000/well in 96-well plates were incubated in media with or without the addition of BRAFi, MEKi and MF-438, administered either alone or in combination for 7 days. The dose–response curves were defined with KaleidaGraph software. Three independent experiments in duplicate were performed.

For other experiments, M14, M14-R, A375, Mel 29 and Mel 66 cell lines were seeded in the presence or absence of BRAFi/MEKi (10 μM for M14 and M14-R; 1 μM for A375, Mel 29 and Mel 66) or MF-438 (1 μM). After 96 h of drugs exposure, cells were washed with Phosphate Buffered Saline (PBS) (Sigma, St. Louis, MO, USA) and harvested for mRNA isolation, WB and IF analyses. The size of spheroids was evaluated as described in Pisanu et al. 2017.

### Quantitative real-time PCR (qRT-PCR) analyses

For qRT-PCR total RNA was isolated with Trizol Reagent (Life Technologies, Gaithersburg, MD, USA) according to the manufacture’s guidelines. RNA was digested with DNAase I (Invitrogen, Carlsbad, CA, USA) and reverse-transcribed into cDNA using High Capacity RNA-to cDNA Kit (Applied Biosystems, Life Technologies, Gaithersburg, MD, USA). Quantitative RT-PCR was performed using SYBR green detection (Applied Biosystem, Life Technologies, Gaithersburg, MD, USA) and the ∆∆Ct method for relative quantification. Expression of β-actin was used as internal control.

The primers used for individual genes are listen in Additional file 1: Table S1.

### Western blot analyses

Protein expression assays were performed as described in Fattore et al. 2015 [39]. Briefly, cells were lysated in RIPA buffer (Sigma, St. Louis, MO, USA) containing the protease inhibitor cocktail (Hoffman-La Roche Ltd) for total lysate or using NE-PER Nuclear Cytoplasmic Extraction Reagent kit (Pierce, Rockford, IL, USA) to separate nuclear fraction from cytosol and the protein lysates were separated on SDS/PAGE acrylamide gel and transferred on Polyvinylidenedifluoride (PVDF) membranes. Membranes were blotted with different antibodies and developed with ECL western blotting substrate (GE Healthcare Life Sciences Marlborough, MA, USA). The primary antibodies used were the following: anti-GAPDH, anti-tubulin, anti-vinculin (Sigma, St. Louis, MO, USA), anti-SCD1 (Abcam, UK), anti-pAKT, anti-AKT, anti-pERK, anti-ERK (Cell Signaling Technology, Beverly, MA, USA), anti-YAP/TAZ and anti-Laminin A/C (Santa Cruz Biotechnologies, Dallas, Texas, USA). All results (mean ± SD of three independent experiments) were normalized over GAPDH, vinculin or tubulin, as specified, and expressed as fold change relative to density of control protein levels.

### Immunofluorescence analyses and optical microscopy

For immunofluorescence analyses cells were fixed with 4% paraformaldehyde (PFA; Sigma-Aldrich), permeabilized in 0.1% Triton-X100 (Sigma-Aldrich, Milan, Italy), after washing two times with PBS the cells were stained with anti-SCD1, anti-JARID1B (Abcam, UK) and anti-YAP/TAZ (Santa Cruz Biotechnologies, Dallas, Texas, USA) antibody (1:50 dilution) or PBS alone as negative control and incubated at 4 °C overnight. Next day, cells were washed by PBS three times to remove unbound antibodies, then secondary antibody (1:300 dilution) was added in the dark and incubated at room temperature for 1 h. Then cells were stained with Hoechest 33,342 (1:1000 dilution) for 5 min in the dark. Immunofluorescence images and morphology observations of cell lines were performed on Axiocam Camera (Zeiss) digital camera coupled with Zeiss Axiovert optical microscope at 100x and 320x magnification and analyzed using ZEN core software (Zeiss). At least 8–10 fields were randomly captured from each sample.

### Transfections

-Plasmid Transfections. SCD1 DNA transfection was performed with Lipofectamine 2000 (Thermofisher) according to the manufacturer’s instructions. All of the transfection experiments were performed with 500 ng of each plasmid.

-siRNA transfection. We transfected small interfering RNA-targeting SCD-1 (Sigma) or Scramble siRNA (Sigma) into adherent cells using Lipofectamine RNAi MAX Reagents (Invitrogen), according to the manufacturer’s recommendation. After 24 h from transfection, cultures were grown in sphere medium and allowed to form spheroids in presence or not of BRAFi and MEKi.

### Lipid extraction

Lipids were extracted from A375 and M14 cells with a method adapted from the Folch’s procedure [40]. Briefly, about 2 × 10^6^ cells were suspended in 300 μL of MilliQ water (18.2 Ω) and cracked by repeated freezing in liquid nitrogen and-thawing. Cell debris were pelleted by spinning at 11000 rpm for 5 min. Protein content was determined by Bradford assay in 5 μL supernatant. The pellet was suspended by vortex-shaking and spiked with ten nmoles of each d_6_-cholesterol and d98-TG 48:0 as the internal standards (ISTD) to control recovery of lipids, retention time (RT), and to calculate the amounts of cholesterol and fatty acid methyl esters (FAME), respectively. The ISTD mixture contained 0.001% of BHT to prevent lipids autoxidation during processing. Liquid-liquid extraction of lipids was performed with 1 mL of CHCl3/CH3OH 2:1 mixture. The bottom organic phase was transferred to a clean glass tube. The operation was repeated twice and the pooled organic phases were evaporated to dryness under a nitrogen stream. The dried lipid extract was dissolved in 400 μL CHCl3/CH3OH 2:1 mixture and used for the analysis of the profile of FAME.

### FAME derivatization

To profile FA mostly bound in glycerol lipids and phosphoglycerol lipids, 90 μL of crude organic extract were evaporated to dryness and then dissolved in 300 μL of 500 mM KOH in anhydrous CH3OH. To favor the saponification and methylation of bound FA the mixture was incubated at 37 °C under constant shaking for 20 min. To terminate reaction and neutralize the alkaline mixture, 300 μL of 250 mM HCl were added. After vortex mixing, 300 μL K2SO4 (6.7%) and 1 mL hexane:isopropanol (3:2 *v*/v) mixture containing 0.0025% BHT were added and vortexed vigorously. After centrifugation, the lipid enriched upper phase was transferred to an Eppendorf tube and evaporated under nitrogen. The reaction yielded FAME that were separated and detected as described below to establish profiles of bound FA in the lipid extracts.

### Gas chromatography-mass spectrometry

Gas-chromatography coupled to mass spectrometry (GC-MS) was used to quantitate FAME. The dried FAME extract was dissolved in 180 μL of n-hexane. One μL was injected onto the GC-MS equipment (Thermo-Finnigan, Waltham, MA, USA). The chromatographic separation was carried out on a HP-FFAP (crosslinked FFAP, Agilent Technologies, Santa Clara, CA, USA) capillary column (length 50 m, film thickness 0.52 μm). Helium was used as the carrier gas. The initial GC oven temperature was 40 °C and was linearly ramped at 8 °C/min up to 240 °C. The total run time was 60 min. The injector and the GC-MS transfer line were kept at 230 °C and 250 °C, respectively. Total ion chromatograms (TIC) were acquired, and areas of single peaks, corresponding to individual FAME, were integrated with the qualitative analysis software. Identity of the detected FAME was verified by comparison with authentic standards and matched with library spectral data. The nmole amounts of FAME were calculated against the nmole of d31-hexadecanoate methyl ester (d31-C16:0ME) yielded from d98-TG 48:0.

### Tissue samples

Total RNA was extracted from the FFPE samples from 3 normal skin with well-represented melanocytes along the basal layer of epidermis, 10 melanocytic common nevi, 3 dysplastic melanocytic nevi, 12 stage I/II melanomas, 11 stage III/IV melanomas, as described in Fattore et al. [41] (see Additional file 2: Table S2).

### Immunohistochemistry

Formalin-fixed paraffin-embedded tissue sections of archival human tissue samples from the San Gallicano Dermatologic Institute, Istituti Fisioterapici Ospitalieri (IFO) of Rome, Italy and from Istituto Cantonale di Patologia of Locarno, Switzerland (12 melanocytic nevi, 12 stage I/II melanomas, 11 stage III/IV melanomas) were obtained with informed and signed consent, and stained with anti-SCD1 (clone CD.E10). In collaboration with Ospedale Sant’Andrea “Sapienza University of Rome” the immunohistochemical staining was assessed by one pathologist. SCD1 score was determined by positive cell (perinuclear/cytoplasm localization) percentage in tumour tissues. Original magnification X 200.

### Bioinformatics analyses

Human lung data were extracted from the GEO database by using Oncomine bioinformatics tool on Talantov dataset [42]. The data represent the median of SCD1 expression. Survival curves were estimated with the Kaplan-Meier product-limit method and compared by log-rank test, accessing data from cBioPortal bioinformatics tool [43, 44].

### Statistical analyses

All experiments were performed in triplicate and values were calculated as mean ± standard deviation (SD) or expressed as a percentage of controls ± SD. SCD1 mRNA and protein expression in patients was described by median value (used as cut-off). Differences between two groups were determined using Anova, two-tailed Mann Whitney or Student’s t-test as specified. Statistical significance was set at *p* < 0.05.

## Results

### SCD1 is potentially useful for discriminating healthy tissue from melanoma

**Fig. 1:**
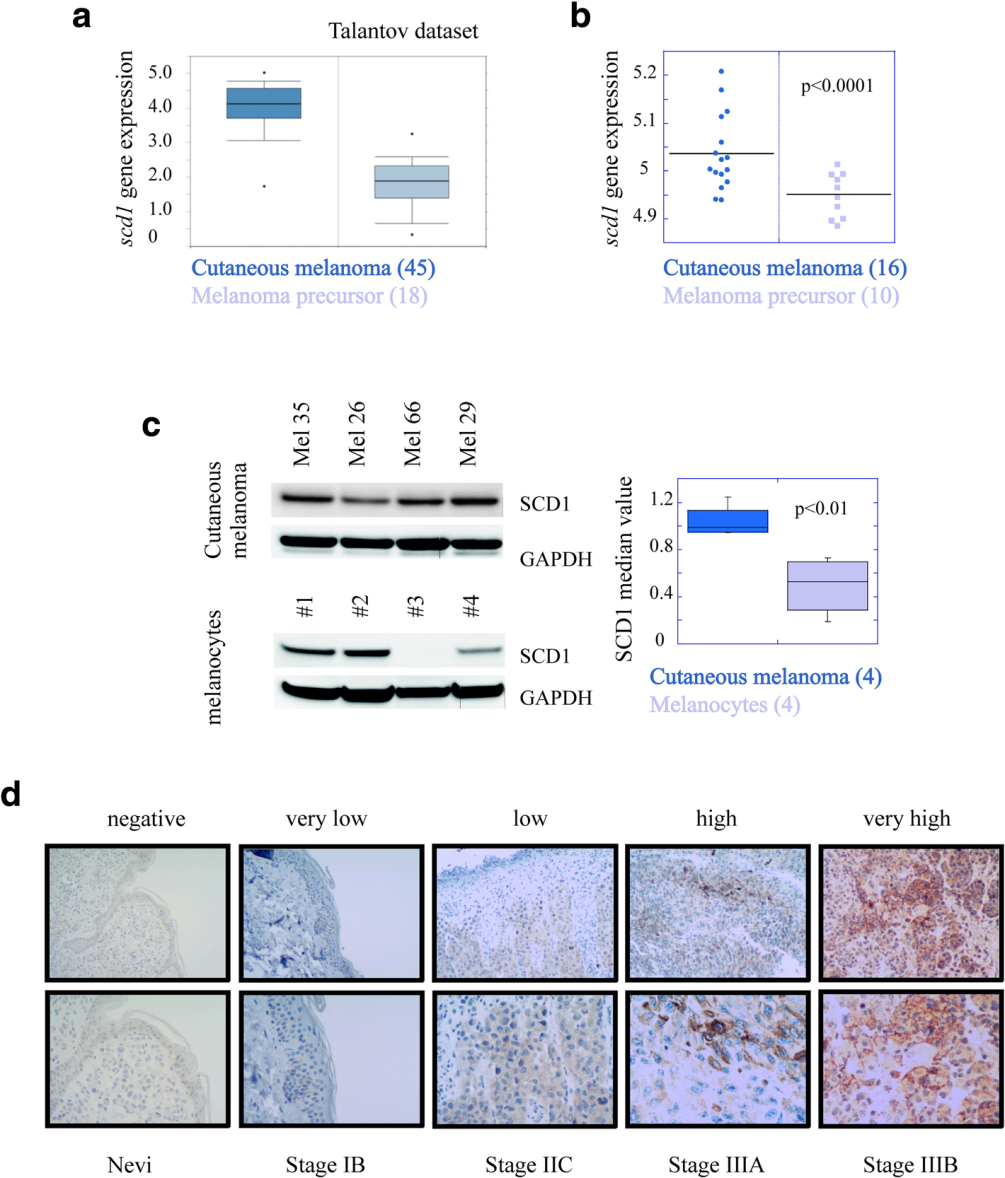
SCD1 is potentially useful for discriminating healthy tissue from melanoma. **a**) Geo Skin Cutaneous Melanoma Talantov dataset was analyzed for the expression of SCD1 by using Oncomine tool. Boxplot: cutaneous melanoma (*n* = 45); melanoma precursor (*n* = 18); **b**) mRNA expression of SCD1 was determined by qRT-PCR analyses in melanoma patients affected by different tumor stage. The samples are grouped for SCD1 gene by stage in melanoma precursor (*n* = 10) and cutaneous melanoma at different stage (*n* = 16); **c**) Western blotting analysis of SCD1 protein in four primary cell lines isolated from patients affected by cutaneous melanoma at different stage (upper panel) and four cell lines obtained from non tumoral tissue (bottom panel). Mel 26 stage IB; Mel 35 stage IIC; Mel 66 IIC and Mel 29 stage IIIC. On the right boxplots represent the quantification of SCD1 levels expressed as median value (fold-change = 1.9, *p* = 0.01); **d**) Representative images showing cellular variability for IHC staining of SCD1 protein in melanoma patients. Magnification 200X (upper) 400X (bottom)

We first sought to determine the diagnostic relevance of SCD1 by analysing RNA levels in melanoma patients. Bioinformatics analyses performed from data extracted from publicly available cancer gene expression dataset (Talantov) [45] using the Oncomine tool (https://www.oncomine.org) demonstrated that SCD1 expression discriminates melanoma from non-tumoral samples (fold change = 3.4, *p* < 0.001) (Additional file 3: Figure S1a). Likewise, SCD1 levels were significantly higher in melanoma than in melanoma precursor lesions (fold change = 4.6, *p* < 0.001) (Fig. 1a). These findings were confirmed in tissues from an independent set of patients by qRT-PCR (Fig. 1b and Additional file 3: Figure S1b) and in a set of four primary cell lines isolated from the same patients by western blot analyses (Fig. 1c). Furthermore, IHC analyses performed on human tissues obtained from melanocytic nevi and melanoma (stage I-IV) showed that SCD1 expression levels increased in parallel with disease progression (*p* < 0.016, Mann Whitney U Test) (Fig. 1d and Additional file 3: Figure S1c). It is important to note that, while the Oncomine tool does not provide information on BRAF status in the Talantov dataset, samples analysed in Fig. 1b, c, d, Additional file 3: Figure S1b and Figure S1c were obtained from BRAF mutated tumours.

Finally, we evaluated the relationship between SCD1 expression and overall survival interrogating the TCGA cohort (http://www.cbioportal.org/). As illustrated in Additional file 3: Figure S1d, elevated expression of SCD1 was associated with shorter survival (Log rank *p* = 0.006), regardless of BRAF mutational status. Based on these data, SCD1 may be a novel prognostic factor in melanoma. However, since BRAF mutated melanomas are the only subset in which a target therapy has been approved, we focused subsequent studies in this subset.

### Higher SCD1 expression correlates with increased stemness and drug resistance

**Fig. 2:**
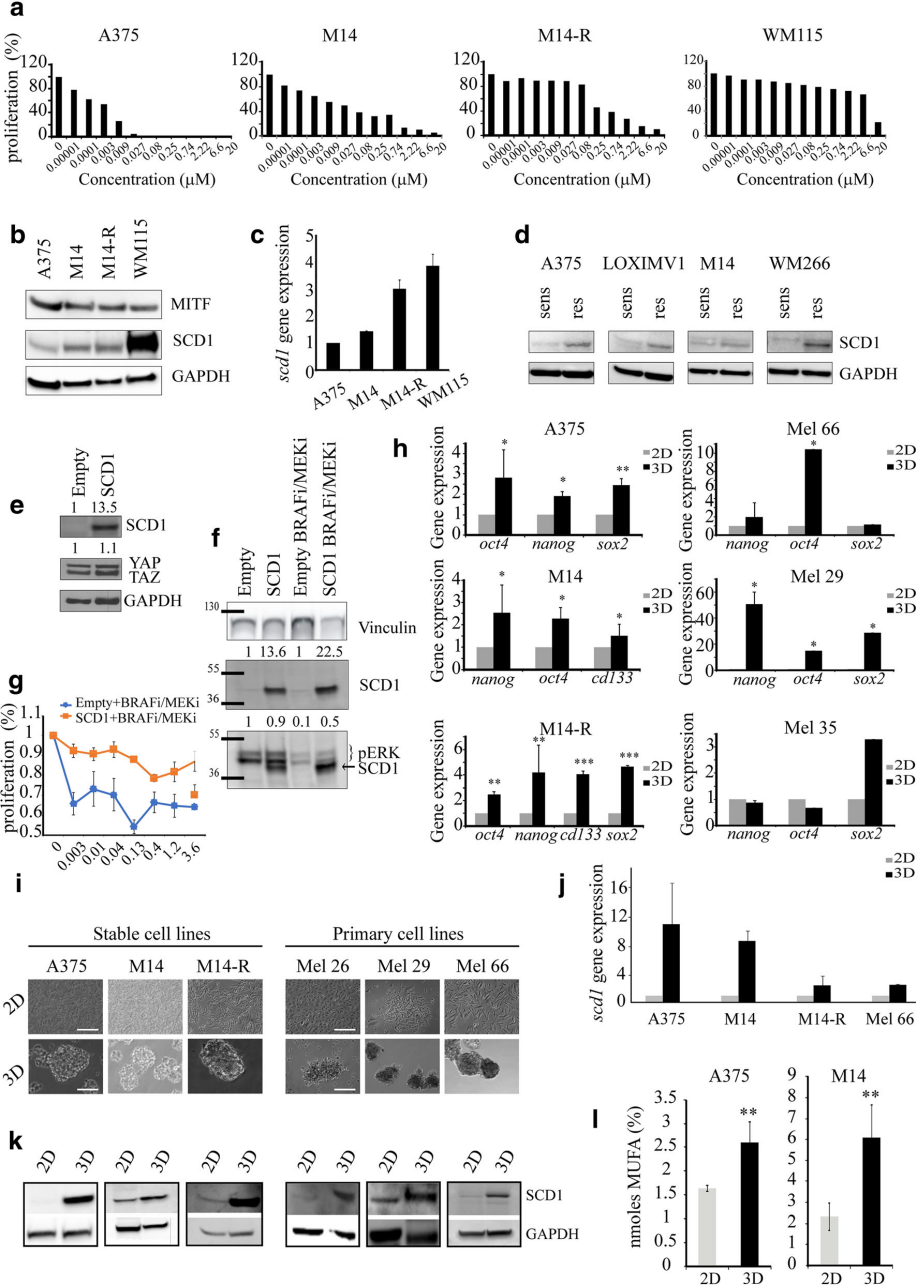
Higher SCD1 expression correlates with increased stemness and drug resistance. **a**) Evaluation of proliferation performed on A375, M14, M14-R, WM115 by MTT assay after 72 h of vemurafenib exposure; **b**) MITF and SCD1 protein expression examined in BRAF-mutated melanoma stable cell lines (A375, M14, M14-R, WM115) grown in adhesion by WB; **c**) qRT-PCR analyses performed on A375, M14, M14-R and WM115 in basal condition; **d**) Western blotting analysis of SCD1 in A375, LOXIMV1, M14 and WM266 sensitive and their counterpart resistant to vemurafenib; **e-f**) Western blotting performed on A375 transfected with empty or SCD1 vector in adherent conditions and treated or not with MAPKi combination (50 + 50 nM) for 48 h. Densitometric analyses relative to SCD1, pERK and YAP/TAZ protein levels were expressed as a fold-change relative to empty (CTRL); **g**) Evaluation of BRAFi/MEKi effects on proliferation performed on 2D A375-transfected with empty or SCD1 vector and treated for 72 h. The dose–effect curves shows a decreased sensitivity of A375 SCD1-overpressing compared to this of A375-transfected with empty vector; **h**) Stemness gene expression in stable (left panel) and primary (right panel) cell lines by qRT-PCR; **i**) Representative images of melanoma cell lines grown in 2D and 3D condition taken on day 4. Scale bars: 50 μM (3D) and 100 μM (2D); **j**) Gene expression of SCD1 performed on 3D and 2D cultures by qRT-PCR analyses. The results indicate an enrichment of SCD1 mRNA expression in 3D spheroids compared with 2D parental cells; **k**) Western blotting analysis of SCD1 protein in a large panel of melanoma stable and primary cell lines grown in 3D and 2D conditions; **l**) MUFA levels in A375 and M14 cell lines analysed by GS/MS in 2D and 3D cultures. Data represent the means and SD of 3 independent experiments and are statistically significant if *p < 0.05 (ANOVA test)

Having documented that SCD1 expression increases with melanoma progression, we hypothesized that SCD1 may be associated with resistance to targeted agents directed against BRAF and MEK. To verify this hypothesis, we first tested the expression of SCD1 in stable melanoma cells lines with different sensitivity to vemurafenib (A375 (IC50 value 0.002 μM), M14 (IC50 value 0.13 μM), M14-R (IC50 value 0.25 μM) and WM115 (IC50 value 2.59 μM)), previously evaluated by MTT assay (Fig. 2a). Western blotting analyses revealed an increase of SCD1 expression in vemurafenib-resistant cells versus sensitive cells, and a similar pattern was recorded at the mRNA level (Fig. 2b-c).

To confirm the supposed connection between SCD1 and resistance to BRAF inhibition, we compared SCD1 expression in four stable resistant cell lines obtained (described in the materials and methods section) and their sensitive counterparts. With this approach, we observed a 2-fold increase in SCD1 levels in resistant cells, thus suggesting that SCD1 sustains resistance to BRAF inhibition (Fig. 2d).

To further corroborate the involvement of SCD1 in reducing the effectiveness of drugs directed against BRAF and MEK we selected the most sensitive cell line to vemurafenib (A375) expressing SCD1 at lower level and verified the sensitivity to BRAF/MEK inhibitors combination after enforced expression of SCD1 (Fig. 2e-g). Interestingly, we observed that while the BRAF/MEK inhibitors combination (50 nM, 1:1) was able to strongly decrease pERK levels, ectopic expression of SCD1 restored pERK levels (Fig. 2f) and mitigated inhibition of cell proliferation (Fig. 2g) thus suggesting that SCD1 activity may promote drug resistance.

On this basis and considering our previous results that delineated the role of SCD1 in: i) maintaining the CSC compartment in lung cancer [27], and ii) inducing chemotherapy-resistant features [28], we hypothesized that lipid metabolic reprogramming may be also a distinctive feature of melanoma CSCs, and that this phenomenon installs therapeutic resistance.

Based on their ability to grow as 3D spheroids, we isolated a highly tumorigenic cell subpopulation characterized by an enrichment of markers associated with stemness such as *oct4, nanog, cd133, sox2* for A375, M14 and M14-R stable and Mel 66, 29, primary BRAF-mutated cells lines (Fig. 2h). This stem-like phenotype in 3D (Fig. 2i) was consistent with an up-regulation of SCD1, both at the transcript (Fig. 2j) and protein expression (Fig. 2k), and with enhanced SCD1 activity evaluated by fatty acid methylesters (FAME) profile by GS/MS. Indeed, 3D cultures exhibited an increased fraction of unsaturated fatty acids compared to 2D cultures in two stable cells analysed (*p* ≤ 0.001) (Fig. 2l).

Collectively these results indicate that an increased expression and activity of SCD1 in the CSC subpopulation may be involved in therapeutic resistance to targeted therapy in melanoma.

### SCD1 expression is able to predict the response of BRAF-mutated-melanoma cells to targeted agents

**Fig. 3:**
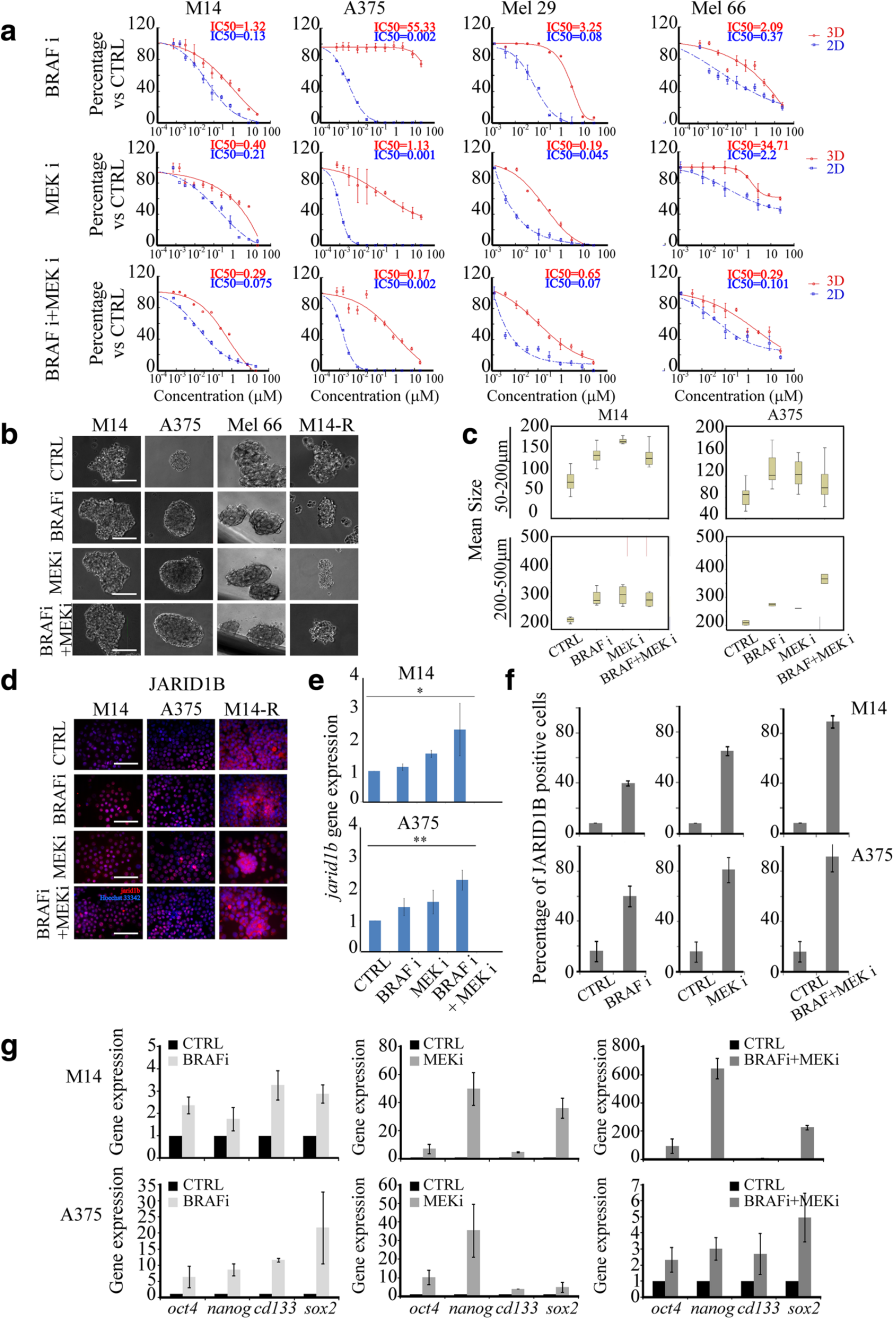
SCD1 expression is able to predict the response of BRAF-mutated-melanoma cells to targeted agents. **a**) Single-cell suspensions of melanoma cell lines were seeded onto a 96-plate ultra low attachment in sphere medium (3D) or in 96-plate cultured in RPMI-1640 (2D). Cell cultures treated with increasing concentrations of BRAF inhibitor (BRAFi) or MEK inhibitor (MEKi) (0.07–20 μM) alone or in simultaneous combination. After 7 days of treatment the sphere-forming efficiency (%) of 3D cancer cells was compared to untreated cells. In parallel the proliferation (%) of 2D cancer cells was compared to control (CTRL). Inset shows the evaluation of drug effects (IC50 value in 3D (red) and in 2D (blu)) performed on melanoma cell lines by Calcusyn software; **b**) Representative images of sphere formation of first generation taken on day 4. Scale bars: 50 μm. Single-cell suspensions of M14, A375, Mel 66 and M14-R cell lines were seeded in sphere medium and simultaneously treated with BRAF and MEK inhibitors alone or in combination for 4 days; **c**) Morphometric analysis of spheroids from treated or untreated M14 and A375. The median value plotted in boxplots showed that treated cultures were characterized by a higher size of spheroids compared to the untreated cultures; **d**) Immunofluorescence analyses on JARID1B expression were performed on M14, A375 and M14-R spheroids after 96 h of exposure to BRAF or MEK inhibitors and their combination; Scale bars: 50 μm; **e**) mRNA expression of *jarid1b* was determined by qRT-PCR after 96 h of drugs exposure. *Jarid1b* results upregulated by BRAF/MEK inhibitors on M14 and a375 cell lines. All data represent the means and SD of 3 independent experiments and are statistically significant if *p* < 0.05 (Anova test); **f**) Percentage of JARID1B positive cells treated with BRAF and MEK inhibitors and their combination; **g**) Stemness markers analysed on M14 and A375 cell lines after BRAF plus MEK inhibitors by qRT-PCR

Given that the clinical role of the MAPKi is widely documented in melanoma, we first tested whether BRAF and MEK inhibitors possess a differential antitumor activity between CSCs and their more differentiated counterparts. Thus, different doses (0–20 μM) of vemurafenib and binimetinib were administered either alone or in combination, in M14 and A375 stable cell lines and in Mel 29 and Mel 66 primary melanoma cell lines growing under 3D and 2D conditions. As illustrated in the dose-response curves presented in Fig. 3a, single and combination therapy were significantly more efficient against cells growing in 2D cultures compared with cells cultured under 3D conditions (see IC50 inserted in Fig. 3a). This observation, coupled with evidence that SCD1 expression levels are higher in 3D vs 2D cultures, suggests that enhanced SCD1 expression may be responsible for the therapy-resistant phenotype of melanospheres.

Remarkably, we found that BRAF and MEK inhibitors, as well as their combination, resulted in an increased compactness and size of spheroids (Fig. 3b-c).

Given that this finding suggested a paradox effect of vemurafenib and binimetinib, namely a potential enrichments of CSCs, we further verified drug-induced increase of melanoma CSCs content by analysing JARID1B (a member of the histone 3 K4 demethylase family), which is an established marker of melanoma CSCs [46, 47].

Immunofluorescence and qRT-PCR analyses consistently documented a marked increase of JARID1B in treated cells (Fig. 3d-e, *P* ≤ 0.02), along with an increased percentage of JARID1B positive cells (Fig. 3f) and overexpression of a set of CSCs markers (*nanog, oct4, sox2 and cd133*) (Fig. 3g, *P* ≤ 0.02).

**Fig. 4:**
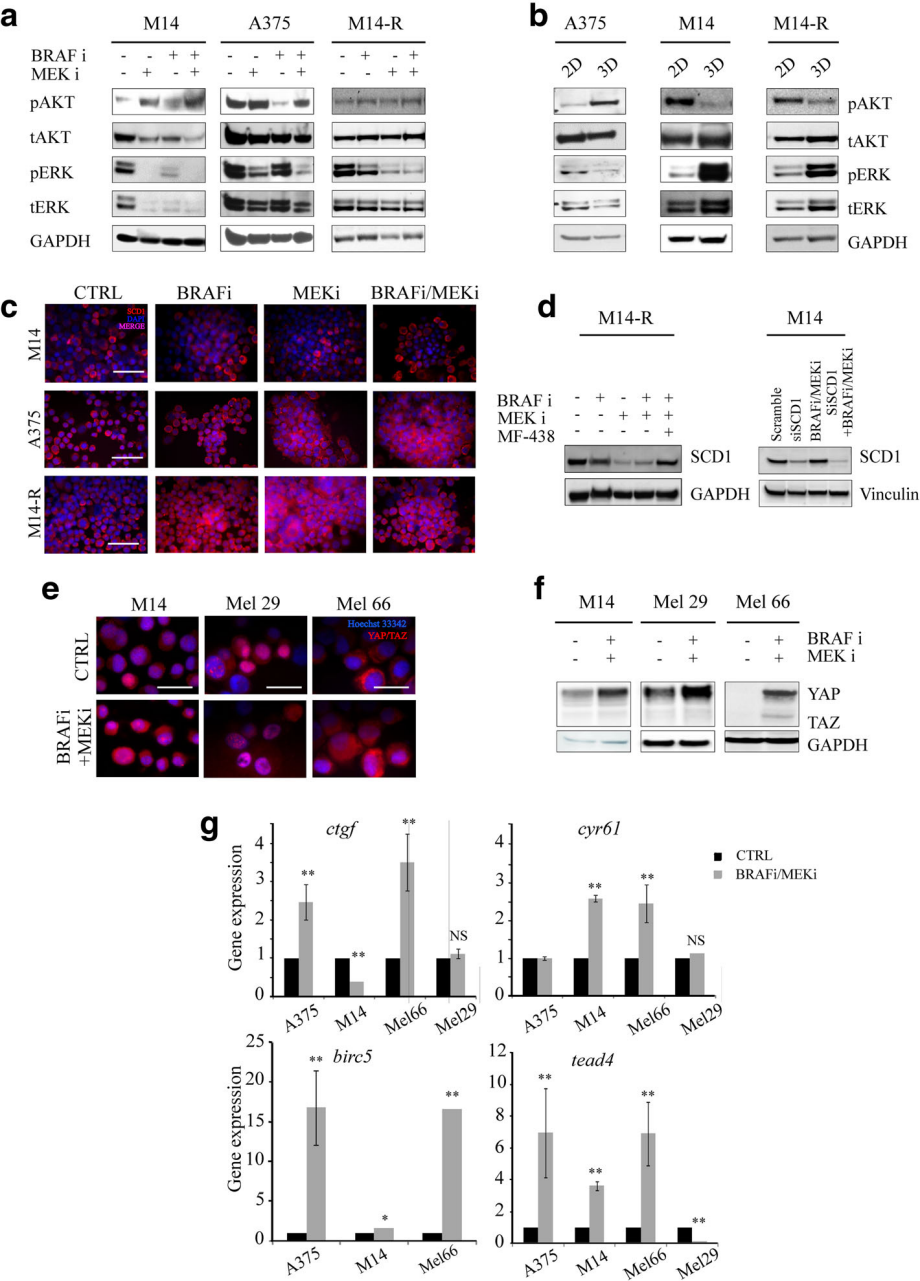
SCD1 expression is able to predict the response of BRAF-mutated-melanoma cells to targeted agents. **a**-**b**) AKT and ERK pathways were examined by WB analyses in protein lysates prepared from M14, A375 and M14-R cells treated with BRAF and MEK inhibitors or their combination (panel **a**) grown in adhesion (2D) and as spheroids (3D) (panel **b**); **c**) SCD1 protein expression performed on fixed M14, A375 and M14-R spheroids after 96 h of treatment with BRAF and/or MEK inhibitors by Immunofluorescence analyses. Scale bar 50 μm; **d**) WB analysis of SCD1 protein expression performed on M14-R and M14 spheroids after 96 h of BRAF and/or MEK inhibitors exposure; **e**) Immunofluorescence analyses on YAP/TAZ expression were performed on fixed M14, Mel 29 and Mel 66 spheroids after 96 h of exposure to BRAF/MEK inhibitors; Scale bars: 10 μm; **f**) Western blotting analysis of YAP/TAZ in M14, Mel 29 and Mel 66 spheroids after BRAF/MEK inhibitors exposure; g) YAP/TAZ downstream target *ctgf*, *cyr61*, *birc5* and *tead4* expression in A375, M14, Mel 29 and Mel 66 by qRT-PCR analyses

As functional evidence pointed to AKT and ERK hyper-activation as a strategy melanoma cells evolve to tolerate prolonged BRAF and MEK inhibition [48, 49], we evaluated total and activated (phosphorylated) forms of AKT and ERK (pAKT and pERK) in untreated and treated spheroids. Western blot analysis did not reveal any clear increase of these proteins under treatment (Fig. 4a). Likewise 3D-cultured cells did not show increased levels of pAKT and pERK compared with their counterparts growing in adhesion (Fig. 4b). Consequently, intrinsic resistance of CSCs to targeted agents seems to be independent from AKT and ERK activation, but rather sustained by SCD1 activity. These findings indicate that while BRAF and MEK inhibitors efficiently eliminate more differentiated melanoma cells, they are ineffective against melanoma CSCs and expand the CSCs pool, in a process that is unrelated to the activation of effectors acting downstream or laterally the MAPK pathway.

Once having excluded the involvement of some established mechanisms of drug resistance in our cellular models, we verified whether the drug-resistant phenotype was related to SCD1. Nevertheless, immunofluorescence and western blotting did not reveal any significant changes in SCD1 expression and in MUFA levels in melanoma cell lines growing as spheroids treated with vemurafenib, binimetinib or both agents versus untreated cells (Fig. 4c-d and data not shown).

Reasoning that SCD1-mediated drug resistance at the CSC level may be related to the control it operates on established stemness-associated molecular signalling, we specifically investigated the Hippo transducers YAP/TAZ in our models. Indeed, experimental evidence points to SCD1 as an emerging controller of YAP/TAZ activity that, in turn, installs CSC traits [26]. We observed an activation of YAP/TAZ in melanoma CSCs treated with BRAF and/or MEK inhibitors, as documented by an increase of YAP/TAZ at the protein level in stable and primary cell lines (M14, Mel 66, Mel 29) (Fig. 4e-f), coupled with the increase of YAP/TAZ target genes such as *ctgf, birc5, cyr61 and tead4* (Fig. 4g). These findings are consistent with a previous study suggesting YAP and TAZ as BRAF inhibitors resistance factors [50].

Thus, treatment with MAPKi (both BRAF and/or MEK inhibitors) enriches the CSC pool, through a process that requires SCD1-mediated increased transcriptional activity of YAP/TAZ.

This suggests that melanoma cells with high levels of SCD1 may be insensitive to MAPKi treatment and that SCD1 could discriminate BRAF-mutated melanoma into MAPK-sensitive and -resistant subpopulations.

### SCD1 inhibition efficiently targets melanoma stem cells and reverted their resistance to BRAF and MEK inhibitors

**Fig. 5:**
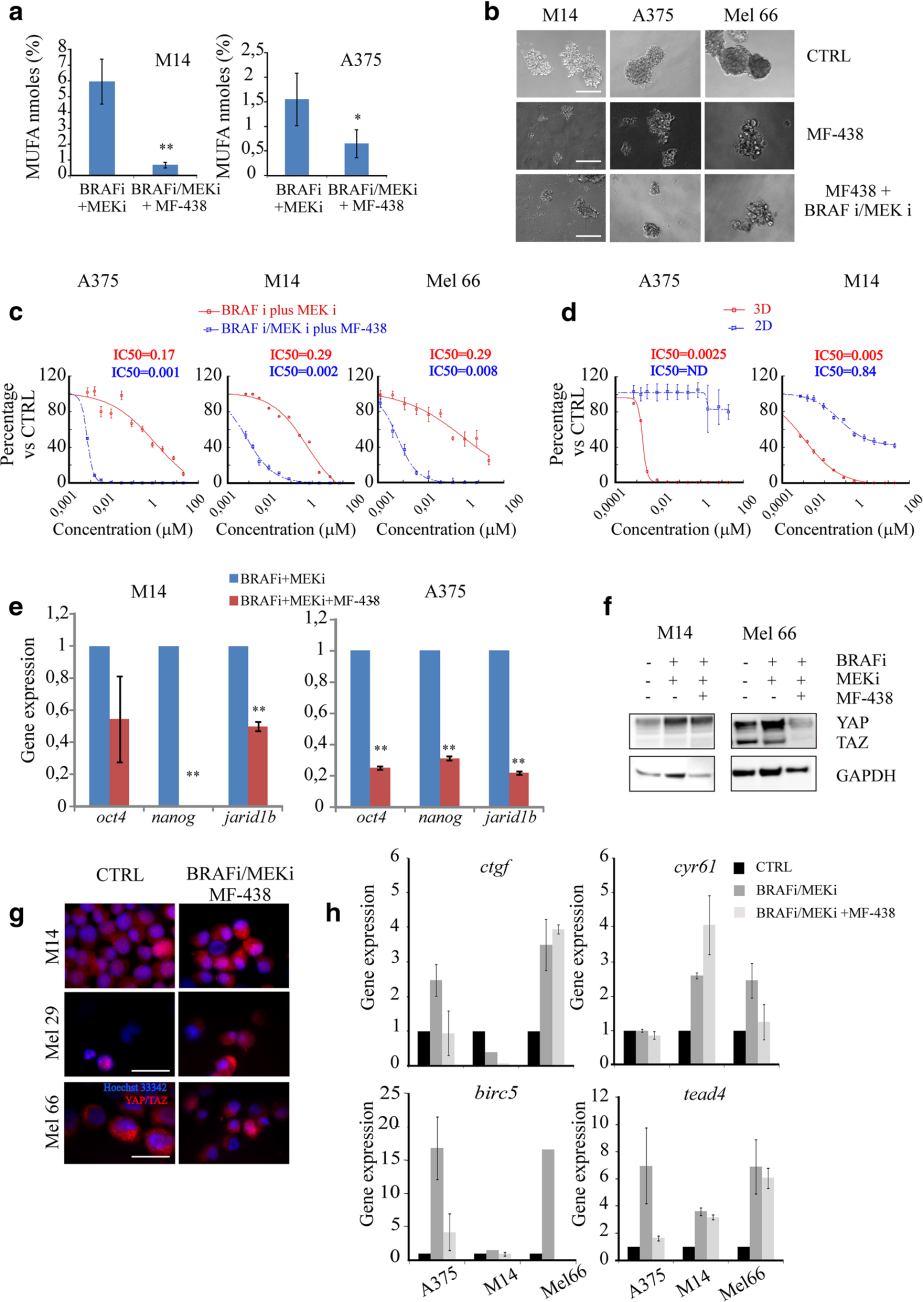
**a**) MUFA levels analysed by GS/MS in M14 and A375 BRAF/MEK plus MF438 treated cells; **b**) 12 Representative images of sphere formation of first generation taken on day 4. Scale bars: 50 μm. 13 Single-cell suspensions of M14, A375 and Mel 66 cell lines were seeded at 1000/well onto a 6-plate 14 ultra low attachment in sphere medium and treated with MF-438 alone or in combination with 15 BRAF/MEK inhibitors for 4 days; **c**) Sphere forming efficiency evaluated on A375, M14 and Mel 16 66 cell lines seeded at 1000/well onto a 96-plate ultra low attachment in sphere medium (3D). Cell 17 cultures treated with increasing concentrations of BRAF and MEK inhibitors (0.07-20 μM) 18 combined or not with MF-438 (0.07-50 μM). After 7 days of treatment the sphere-forming 19 efficiency of 3D cancer cells was compared to untreated cells; **d**) Proliferation assay performed on 20 2D and 3D cultures obtained from A375 and M14 cell lines exposed to MF-438 for 7 days; inset 21 shows the IC50 value calculated in 3D culture treated with BRAF/MEK and or BRAF/MEK plus 22 MF-438 (panel **c**) and IC50 3D vs 2D condition (panel **d**); **e**) Stemness markers (oct4, nanog, 23 jarir1b) analysed on M14 and A375 melanoma cells after BRAF/MEK plus MF-438 inhibitors by 24 qRT-PCR; **f**) Western blotting analysis of YAP/TAZ in M14 and Mel 66 spheroids treated with 25 BRAF, MEK or BRAF/MEK plus MF-438 for 96 hours; **g**) Immunofluorescence analyses of YAP/TAZ after BRAF/MEK inhibitors plus MF-438 performed on M14 and Mel 66 cell lines. 2 Scale bar 10mm; **h**) YAP/TAZ downstream target analysed after MF-438 combined with BRAF and 3 MEK inhibitors in A375, M14 and Mel 66

We have previously reported on the ability of MF-438 to efficiently inhibit SCD1 function. To address the anti-CSCs properties of MF-438, 3D melanoma cell cultures were exposed to MF-438 given as single-agent or in combination with vemurafenib and binimetinib. Consistent with the preferential activation of SCD1 in the CSCs pool, its inhibition in M14 and A375 decreased MUFA levels (Fig. 5a), hindered sphere-forming efficiency when given as single treatment (Fig. 5b), and overcame the intrinsic resistance of spheroids to BRAF and MEK inhibitors (Fig. 5c). Next, we compared the antitumor activity of MF-438 in 3D cultures versus their differentiated counterparts. Figure 5d shows that treatment with MF-438 reduced cell viability of CSCs, while resulting largely ineffective against non-CSCs. These lethal effects were accompanied by decreased expression levels of the stem cell markers *oct4, nanog* and *jarid1b* (Fig. 5e).

**Fig. 6:**
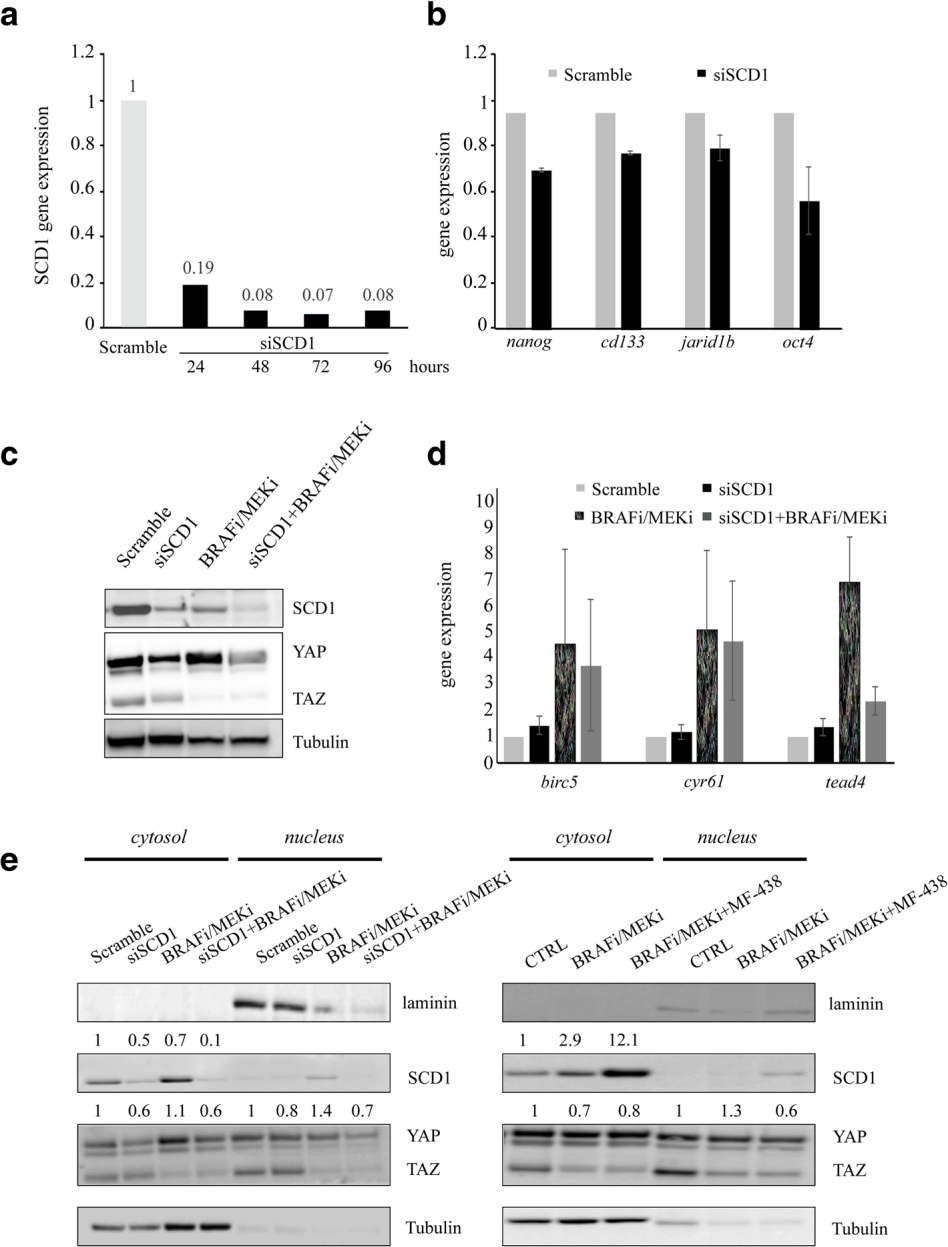
SCD1 inhibition efficiently targets melanoma stem cells and reverted their resistance to MAPK inhibitors. **a**) Efficiency of silencing of SCD1 analyzed by qRT-PCR performed on M14 cells grown in 2D for 96 h; **b**) Gene expression of *nanog, cd133, jarid1b* and *oct4* after SCD1 silencing in M14 spheroids determined by qRT-PCR; **c**) Representative western blotting analysis of total lysates obtained from M14 silenced and treated with BRAFi/MEKi showing SCD1 and YAP/YAZ protein expression; **d**) Gene expression analyses of YAP/TAZ gene targets performed on M14 spheroids SCD1 silenced and treated with BRAFi/MEKi combination. The results confirmed that SCD1 inhibition by silencing partially reverts the enrichment of *YAP/TAZ gene targets* induced by MAPKi exposure; **e**) Western blotting of nuclear and cytosolic fractions obtained from M14 spheroids treated with BRAFi/MEKi in presence of SCD1 silencing (left panel) or simultaneously treated with MF-438 (right panel). Densitometric analyses of western blotting showed as a fold-change vs relative CTRL

Moreover, we observed that the triple targeting of BRAF-MEK-SCD1 in resistant spheroids reduced YAP/TAZ protein levels, their nuclear accumulation and expression of YAP/TAZ target genes (Fig. 5f-h). To confirm that the observed biological effects on melanoma CSCs are related to selective SCD1 targeting we silenced SCD1 and, after checking the silencing efficiency (Fig. 6a) we analysed the mRNA expression levels of the stem cell markers *nanog, cd133, jarid1b and oct4.* Consistently with our hypothesis, a decrease in their levels was observed (Fig. 6b). Importantly, SCD1 silencing led to a downregulation of YAP/TAZ expression at the protein level mostly in BRAF/MEKi-treated spheroids (Fig. 6c). Moreover, mRNA levels of *birc5* and *tead4* genes which were increased after BRAF/MEK inhibitor treatment, decreased upon SCD1 silencing (Fig. 6d).

Finally, to better understand the mechanisms behind changes in YAP/TAZ activity following drug treatments we also determined changes in YAP/TAZ cellular distribution upon MAPKi treatment, SCD1 inhibition by RNA silencing and pharmacological treatments or their combinations. This was carried out by analysing their expression levels in the cytosolic and nuclear compartment respectively (Fig. 6e). Overall, we observed that MAPKi exposure induced a slight increase of YAP nuclear localization accompanied by a parallel decrease in the cytosol fraction. In contrast SCD1 inhibition reduced YAP levels both in the nucleus and in the cytosol.

Collectively, our findings confirmed that SCD1 activation denotes a metabolic route preferentially activated in melanoma CSCs, and that its abrogation preferentially killed this subpopulation.

Overall, our data support the role of SCD1 as a promising therapeutic target in combination with MAPKi in BRAF mutated melanoma and suggest a possible function as diagnostic and prognostic marker.

## Discussion

The advent of molecular targeted agents dramatically changed the treatment landscape of advanced melanoma. Nevertheless, melanoma cells adapt to the block of BRAF and MEK, becoming able to thrive even under pharmacological pressure. Thus, achieving a deeper understanding of the forces feeding therapeutic resistance to current pathway-focused inhibitors is of utmost importance. In the present study, we investigated how melanospheres react when exposed to routinely used molecularly targeted agents, namely the BRAF inhibitor vemurafenib and MEK inhibitor binimetinib. Overall, our findings indicate that: i) BRAF and MEK inhibitors are unable to eliminate MSCs, which however do not rely on AKT and/or ERK activation to endure vemurafenib- and binimetinib-induced death stimuli, ii) increased activity of SCD1, the rate-limiting enzyme in the formation of monounsaturated fatty acids, enables melanoma CSCs to survive BRAF and MEK inhibition, iii) this process is related to the control SCD1 operates on YAP/TAZ, and iv) pharmacological inhibition of SCD1, achieved with MF-438, selectively killed melanoma CSCs and partly restored sensitivity to the combination of vemurafenib and trametinib. To our knowledge, this is the first report describing the involvement of lipid metabolism in sustaining therapeutic resistance in melanoma CSCs. Moreover, data herein presented open up a novel therapeutic scenario, envisioning the inhibition of specific metabolic routes to erase melanoma-initiating cells. Moreover, SCD1 inhibition was found to selectively target cancer cells, while sparing non-transformed cells. This is of great relevance for therapeutic purposes, as the potential activity of SCD1 inhibition might not be counterbalanced by excessive side effects.

Metabolic reprogramming is considered a hallmark of cancer. Metabolic changes occurring upon malignant transformation are instrumental to cope with genetically deregulated proliferative signalling, and to withstand hostile environmental conditions such as hypoxia and low availability of nutrients. While glucose and glutamine pathway alterations have been recognized as central metabolic changes since the earliest biochemical studies, the contribution of lipids and cholesterol pathways is still underestimated. Nevertheless, as evidence accumulate, lipid reprogramming is gaining popularity given that alterations in lipid composition (e.g. content of saturated versus unsaturated fatty acids) is intimately tied to protein dynamics and membrane fluidity. In particular, monounsaturated fatty acids, derived from saturated fatty acids by the action of SCDs, have been associated with the acquisition of malignant features [51, 52]. However, how lipid metabolism is concatenated with CSC fate remains un understudied domain of stem cell biology [53]. Indeed, since the discovery of CSCs two decades ago, characterization efforts have mostly been oriented toward blocking the so-called stem cell pathways (e.g. Notch, Hedgehog, TGF-β), and to interfere with the molecular network deputed to protect their genome in the attempt of reverting chemo-resistance [54]. Even though the study of lipid metabolism in CSC is still in its infancy, recent studies are beginning to shed light on a novel regulatory force [55]. Parallel with the appreciation of metabolic avenues that operate in CSCs, the inhibition of specific metabolic functions has been proposed for therapeutic purposes. For instance, activation of the mevalonate pathway, which is responsible for the synthesis of cholesterol, has been found to endow breast cancer cells with stem cell traits [56]. Consistently, the targeting of HMG-CoA reductase, the rate-limiting enzyme of the mevalonate cascade, achieved with cholesterol-lowering agents (statins), resulted effective against breast cancer stem cells. Regarding SCD1, previous studies pointed to SCD1 activity as a novel player involved in maintaining stemness in ovarian and lung cancer cells [26–31]. Our study adds a further piece to the puzzle, providing a nexus between lipid alterations, stem cell pathway (YAP/TAZ) and targeted therapy resistance at the CSC level. We envision that two major questions should deserve increased attention in future studies attempting to delineate the metabolic landscape of CSCs, and its connection with therapeutic resistance. First, the metabolic demand of CSCs plausibly varies in relation to the switch from quiescence to proliferation and vice versa, thus adding an element of dynamicity that deserves tailored investigations. Second, the molecular output of lipid reprogramming is still unclear. Even though pioneering studies are beginning to connect lipid metabolism to CSCs via intermediate molecular cascades (e.g. the Hippo pathway), we foresee the existence of a broader network of canonical signal transduction pathways whose activity, and consequent impact on CSC properties, can be tuned by alterations in lipid content.

## Conclusions

In this study, we provide evidences that melanoma CSCs are able to tolerate the pharmacological inhibition of BRAF and MEK, a process sustained by the increased function of SCD1. This, in turn, enhances the transcriptional activity of the Hippo transducers YAP/TAZ. Consistently, inhibition of SCD1 elicits lethal effects and override the intrinsic resistance to BRAF and MEK inhibitors that characterizes melanoma CSCs.

## Additional files

Additional file 1: **Table S1.** The primers used for individual genes. (DOCX 25 kb)

Additional file 2: **Table S2.** Summary of the Clinicopathologic Characteristics. (DOCX 47 kb)

Additional file 3 **Figure S1.** a) Geo Skin Cutaneous Melanoma dataset was analyzed for the expression of SCD1 by using Oncomine tool. The samples are grouped in normal (skin *n* = 7) and cutaneous melanoma (45). The median value is statistically different (*p* < 0.001); b) SCD1 gene expression by qRT-PCR in melanocytes (*n* = 3) vs cutaneous melanoma (*n* = 21). The data represent the median value of log_2_ΔCt. The results are significant (*p* < 0.001); c) Quantification of SCD1 expression in tissue of melanocytic nevi (*n* = 12) and cutaneous melanoma (*n* = 21). SCD1 level expressed as median value was present at different expression levels (> 70% at late stages, (> 10 - < 70% at intermediate stages, ≤ 10% at early stages). Comparison of two groups by Mann Whitney U test resulted statistically significant (*p* < 0.016); d) Kaplan-Meier curves indicating SCD1 mRNA expression analyzed by using cBioPortal tool. Red curve represents patients group in which SCD1 gene is overexpressed, while blue curve represents patients expressing low SCD1 content. (TIF 3558 kb)

## Abbreviations

CSCs: Cancer Stem Cells
FAME: fatty acid methylesters
GS/MS: Gas chromatography-mass spectrometry
MAPKi: mitogen-activated protein kinase inhibitors
MF-438: 2-methyl-5-(6-(4-(2-(trifluoromethyl)phenoxy)piperidin-1-yl)pyridazin-3-yl)-1,3,4-thiadiazole
MTT: 3-(4,5-dimethylthiazol-2-yl)-2,5-diphenyltetrazolium bromide
MUFAs: Monounsaturated Fatty Acids
SCD1: Stearoyl-CoA Desaturase 1
SFAs: Saturated Fatty Acids
SFE: Sphere Forming Efficiency
TAZ: transcriptional coactivator with PDZ-binding motif
YAP: Yes-associated protein
