# Supplementary material for: Inhibition of Stearoyl-CoA desaturase 1 reverts BRAF and MEK inhibition-induced selection of cancer stem cells in BRAF-mutated melanoma
Source: J Exp Clin Cancer Res. 2018 Dec 17;37:318. doi: 10.1186/s13046-018-0989-7 (PMC6298024; doi:10.1186/s13046-018-0989-7)
Supplement: Supplementary file 1 — Table S1. The primers used for individual genes. (DOCX 25 kb) [file 13046_2018_989_MOESM1_ESM.docx]

| *Oct4* | -Forward 5′- TGGGATATACACAGGCCGATG -3′  -Reverse 5′- TCCTCCACCCACTTCTGCAG -3′ |
| --- | --- |
| *Nanog* | -Forward 5′- TACCTCAGCCTCCAGCAGATG -3′  -Reverse 5′- CCTTCTGCGTCACACCATTG -3′ |
| *SCD-1* | -Forward 5′- GCAGG-GGGAACCAGTATGA -3′  -Reverse 5′- TTCTTACACGACCACCACCA -3′ |
| *jarid1b* | -Forward 5′- AGCAGACTGACCGAA GCTCA -3′  -Reverse 5′- AATTCCATCTCGCTT CCCTC -3′ |
| *sox2* | -Forward 5′- CAC CCC TGG CAT GGC TCT T -3′  -Reverse 5′- GAG CTG GCC TCG GAC TTG A -3′ |
| *cd133* | -Forward 5′- TGG ATG CAG AAC TTG ACA ACG T -3′  -Reverse 5′- ATA CCT GCT ACG ACA GTC GTG GT -3′ |
| *β-actin* | -Forward 5′- GCCGGGACCTGACTGACT -3′  -Reverse 5′- TGGTGATGACCTGGCCGT -3′ |
| *ctgf* | -Forward 5′- AGGAGTGGGTGTGTGACGA-3′  -Reverse 5′- CCAGGCAGTTGGCTCTAATC -3′ |
| *cyr61* | -Forward 5′- CAGGACTGTGAAGATGCGGT -3′  -Reverse 5′- GCCTGTAGAAGGGAAACGCT -3′ |
| *tead4* | -Forward 5′- TTGGAACTGGCTTAGCGCAC -3′  -Reverse 5′- CGTCATTGTCGATGGGCTTG -3′ |
| *birc5* | -Forward 5′- AGCATTCGTCCGGTTGCGCT -3′  -Reverse 5′- TCGATGGCACGGCGCACTTT -3′ |

Supplementary Table 1. The primers used for individual genes.
