# Supplementary material for: Inhibition of Stearoyl-CoA desaturase 1 reverts BRAF and MEK inhibition-induced selection of cancer stem cells in BRAF-mutated melanoma
Source: J Exp Clin Cancer Res. 2018 Dec 17;37:318. doi: 10.1186/s13046-018-0989-7 (PMC6298024; doi:10.1186/s13046-018-0989-7)
Supplement: Supplementary file 2 — Table S2. Summary of the Clinicopathologic Characteristics. (DOCX 47 kb) [file 13046_2018_989_MOESM2_ESM.docx]

| **Year of birth** | **Sex** | **Sample**  **material_type** | **FFPE** | **Diagnosis** | **Stage** | **Ulceration** | **Thickness** | **BRAF**  **status** |
| --- | --- | --- | --- | --- | --- | --- | --- | --- |
| 2001 | F | Back | X | Melanocytic dermal nevus | - | - | - | Not mutated |
| 1975 | F | - | X | Melanocytic dermal nevus | - | - | - | Not mutated |
| 1959 | F | Nose, neck | X | Melanocytic dermal nevus | - | - | - | Not mutated |
| 1971 | F | Left lumbar | X | Melanocytic dermal nevus | - | - | - | Not mutated |
| 1963 | M | Scalp | X | Melanocytic dermal nevus | - | - | - | Not mutated |
| 1985 | F | Left shoulder | X | Melanocytic dermal nevus | - | - | - | Not mutated |
| 1968 | M | Left abdomen | X | Melanocytic dermal nevus | - | - | - | Not mutated |
| - | M | - |  | Melanocytic dermal nevus | - | - | - | Not mutated |
| - | M | - |  | Melanocytic dermal nevus | - | - | - | Not mutated |
| 1934 | M | Right scapula | X | Dysplastic Nevus | - | - | - | Not mutated |
| 1967 | F | Left arm | X | Dysplastic Nevus | - | - | - | Not mutated |
| 1973 | M | Right forearm | X | Dysplastic Nevus | - | - | - | Not mutated |
| 1935 | F | Left tibia | X | Malignant melanoma | pT1a | - | 0.8 mm | Mutated |
| 1972 | M | Right chest | X | Malignant melanoma | pT1b | - | 0.6 mm | Mutated |
| 1973 | M | Right forearm | X | Malignant melanoma | pT2a | - | 1.1 mm | Mutated |
| 1963 | F | Right arm | X | Malignant melanoma | pT1a | - | 0.5 mm | Mutated |
| 1952 | F | Back | X | Malignant melanoma | pT1a | - | 0.85 mm | Mutated |
| 1930 | M | Back | X | Malignant melanoma | pT1a | - | 0.35 mm | Mutated |
| 1927 | F | Right cheek | X | Malignant melanoma | pT1b | - | 0.9 mm | Mutated |
| 1915 | F | Back | X | Malignant melanoma | pT4b | Present | 4.8 mm | Mutated |
| 1936 | M | Right arm | X | Malignant melanoma | pT3a | - | 2.2 mm | Mutated |
| 1925 | M | Left eyelid | X | Malignant melanoma | pT3b | Present | 4 mm | Mutated |
| 1929 | F | Left leg | X | Malignant melanoma | pT4b | Present | 5 mm | Mutated |
| 1967 | F | Left lumbar | X | Malignant melanoma | pT3b | Present | 2.5 mm | Mutated |
| 1947 | M | Back | X | Malignant melanoma | pT4b | Present | 5.5 mm | Mutated |
| 1935 | M | Back | X | Malignant melanoma | pT4a | - | 5.9 mm | Mutated |
| 1932 | M | Back | X | Malignant melanoma | pT3b | Present | 2.2 mm | Mutated |
| 1942 | M | Right eyehole | X | Malignant melanoma | pT3a | - | 4 mm | Mutated |
| 1948 | M | - | X | Malignant melanoma | pT4b | - | 4mm | Mutated |
| 1973 | M | - | X | Malignant melanoma | pT4b | - | 6mm | Mutated |
| 1979 | M | - | X | Malignant melanoma | pT3b | - | 2.5mm | Mutated |
| 1972 | F | - | X | Malignant melanoma | pT1b | - | 0.6mm | Mutated |
| 1952 | F | - | X | Malignant melanoma | pT4b | - | 8mm | Mutated |

Supplementary Table 2: Summary of the Clinicopathologic Characteristics.
